# Functional MRI Signal Complexity Analysis Using Sample Entropy

**DOI:** 10.3389/fnins.2020.00700

**Published:** 2020-07-02

**Authors:** Maysam Nezafati, Hisham Temmar, Shella D. Keilholz

**Affiliations:** ^1^Department of Biomedical Engineering, Georgia Institute of Technology, Emory University, Atlanta, GA, United States; ^2^Neuroscience Program, Graduate Division of Biological and Biomedical Sciences, Laney Graduate School, Emory University, Atlanta, GA, United States

**Keywords:** functional MRI, complexity, entropy, temporal analysis, resting state, computational neuroscience, neuro imaging

## Abstract

Resting-state functional magnetic resonance imaging (rs-fMRI) is an immensely powerful method in neuroscience that uses the blood oxygenation level-dependent (BOLD) signal to record and analyze neural activity in the brain. We examined the complexity of brain activity acquired by rs-fMRI to determine whether it exhibits variation across brain regions. In this study the complexity of regional brain activity was analyzed by calculating the sample entropy of 200 whole-brain BOLD volumes as well as of distinct brain networks, cortical regions, and subcortical regions of these brain volumes. It can be seen that different brain regions and networks exhibit distinctly different levels of entropy/complexity, and that entropy in the brain significantly differs between brains at rest and during task performance.

## Introduction

Resting-state functional magnetic resonance imaging (rs-fMRI) uses the blood oxygenation level-dependent (BOLD) signal to characterize the spontaneous activity of the brain (Biswal et al., 2010). In functional connectivity analysis, correlation is calculated between the time series of different regions of interest (ROI). Analysis of regions with high correlation can aid in the identification of functional networks. However, both within and across networks, fMRI signals can exhibit dynamics that may reflect changes in brain state or mental activity (Chang and Glover, 2010; Hutchison et al., 2013; Keilholz et al., 2013, 2016; Keilholz, 2014). If the complexity or regularity of these fluctuations in the fMRI time-series could be evaluated and analyzed, it might provide insight into general brain activity, brain structures, and brain networks.

Entropy, in general, is defined as a lack of order or predictability in a system. In the context of physiologically time-based signals, entropy is a measure of disorderliness in the time dimension. This can also serve as a parallel for system complexity, as a more complex system will often produce more disorderly signals. The second law of thermodynamics indicates that the maximum entropy is reached when a closed system rests at an equilibrium state, so in order to preserve vital activities, living systems must constantly maintain low-levels of entropy, shying away from equilibrium (Schrödinger, 1945). The human brain is one such living system, and while it maintains a relatively low level of entropy when compared to a system at equilibrium, the complex nature of its various networks’ spontaneous activities can cause a variation in local entropy that reflects differences in the function of those areas. By calculating the entropy of the signal acquired from the brain, these variations can be investigated, and the status of the resting brain can be compared across conditions to understand how the brain adapts during task performance (Bergström, 1969; Singer, 2009).

Establishment of a general algorithm for entropy requires a vast data set. The accurate estimation of the probability distribution function from the limited number of time points obtained in rs-fMRI studies is difficult. Sample entropy (Shalit, 1985), an extension of approximate entropy (Pincus, 1991), is an alternative technique that addresses these issues. The Kolmogorov (1998) complexity model is the basis for approximate and sample entropy (Wang et al., 2014), which can be evaluated even with relatively small data sets (Shalit, 1985; Pincus, 1991). This makes them well-suited for the analysis of rs-fMRI data, where the number of time points is typically relatively small (∼200–1000) compared to the number of voxels.

Previous analyses of the entropy of the BOLD signal have shown that brain signals exhibit various levels of disorder. Wang et al. (2014) performed a comprehensive study on the Sample entropy map of approximately 1000 healthy subjects. They observed a sharp low-high contrast between the neocortex and the rest of the brain, which may indicate the higher mental functions performed by cortex (Wang et al., 2014). Moreover, entropy differs in patients with attention deficient hyperactivity disorder (ADHD) (Sokunbi et al., 2013) and during the administration of different drugs (Ferenets et al., 2007).

Motivated by these findings, we performed a more extensive analysis of entropy to determine the amount of variability present across brain areas and networks. We compared findings from task-based and resting state data from the Human Connectome Project (Van Essen et al., 2012) to characterize how entropy changed across conditions. The results provide further evidence that the entropy of the BOLD signal reflects aspects of the brain’s functional organization and may prove informative about neural processing.

## Materials and Methods

### Data Acquisition

MRI Data was downloaded from the Human Connectome Project (Van Essen et al., 2012). This data came from 100 randomly selected, unrelated individuals, ranging from ages 22 to 36 (54 female – 46 male). One anatomical scan from each individual was used for preprocessing [T1-weighted three-dimensional magnetization-prepared rapid gradient echo (T1w 3D MPRAGE) sequence; TR = 2400 ms, TE = 2.14 ms, TI = 1000 ms, FA = 8°, FOV = 224 mm × 224 mm, voxel size 0.7 mm isotropic] (Milchenko and Marcus, 2013).

In addition, two resting-state functional scans per subject, each approximately 15 min long, were used, with the following parameters: TR = 720 ms, TE = 33.1 ms, FA = 52°, FOV = 208 mm × 180 mm (RO × PE), matrix = 104 × 90 (RO × PE), slice thickness = 2.0 mm; 72 slices; 2.0 mm isotropic voxels, multi-band factor = 8, echo spacing 0.58 ms, with right-to-left (RL) phase encode direction in one scan and left-to-right (LR) phase encode direction in the other (Feinberg et al., 2010; Chen et al., 2015). Two working memory task functional scans per subject, each approximately 5 min long, were used for comparison with the rest scans, also with RL phase encode direction in one scan and LR phase encode direction in the other. The working memory task, described in Barch et al. (2013), involves a version of the N-back task, assessing both working memory and cognitive control in a block format. Each task functional scan includes eight task blocks lasting 25 s as well as four fixation blocks lasting 15 s. Half the task blocks use a 0-back working memory task and the other half use a 2-back working memory task. These blocks are divided into four categories: tools, body parts, faces, and places. To adjust for the shorter length of the task scans, rest scans were truncated to the same length as the task scans.

### Preprocessing Methods

Scans were preprocessed using both FSL 5.0 (Jenkinson et al., 2012) and MATLAB (Mathworks, Natick, MA, United States). First, FSL was used to register anatomical data to the 2 mm Montreal Neurological Institute (MNI) atlas using FMRIB’s Linear Image Registration Tool (FLIRT) (Jenkinson and Smith, 2001; Jenkinson et al., 2002). Then the brain was extracted from the scan using BET, and segmented into gray matter, white matter, and CSF using FMRIB’s Automated Segmentation Tool (FAST) (Zhang et al., 2001). Functional data was then motion correction using MCFLIRT (Jenkinson et al., 2002) and registered to MNI space using FLIRT.

The seven functional networks based on Yeo et al.’s (2011) parcellation method (were discriminated from each other. The mean and the standard deviation of sample entropy for each network were calculated and mapped to that network.

### Entropy Calculation

A combination of home-designed MATLAB codes and the brain entropy mapping toolbox (BENtbx^1^) by Wang et al. (2014) from University of Pennsylvania were used to calculate the entropy for each voxel. The equation for sample entropy is described in BENtbx as follows:

$$B^{m}\,\left(r\right)=\frac{1}{\left(N-m\right)\,\left(N-m-1\right)}\,\sum_{i=1}^{N-m}B_{i}^{m}\,\left(r\right)$$

$$A^{m}\,\left(r\right)=\frac{1}{\left(N-m\right)\,\left(N-m-1\right)}\,\sum_{i=1}^{N-m}B_{i}^{m+1}\,\left(r\right)$$

$$S\,E\,\left(m,r,N,x\right)=-l\,n\,\left[\frac{A^{m}\,\left(r\right)}{B^{m}\,\left(r\right)}\right]$$

The fMRI data for one voxel is considered as *x* = [*x*_1_, *x*_2_, …, *x*_*N*_], in this set “*N*” is the number of repetitions (*N* value is specified based on functional scan length and TR). In sample entropy a series of embedded vectors with m consecutive points are extracted from the data set x: *u*_*i*_ = [*x*_*i*_, *x*_*i*__+__1_, … *x*_*i*__+_*_*m*_*_–__1_], (*i* = 1 to *N*−*m* + 1, *m*: pre-defined dimension which specifies the pattern length). A distance threshold “*r*” is specified (tolerance value) and $B_{m}^{r}\,\left(r\right)$ counts the number of *u*_*j*_ (*j* = 1, to *N*−*m*, and *j* ≠ *i*) whose distances to u_*i*_ are less than *r*, as does, $B_{i}^{m+1}\,\left(r\right)$ for the dimension of *m* + 1. Thus the sample entropy can be measured by averaging across all possible vectors (Wang et al., 2014). A small value for ***m*** results in improvement of sample entropy accuracy so in current study the value of ***m*** was equal to 3. Based on the previous studies the threshold of *r* = 0.6 SD would result in similar values of sample entropy even for different values of ***m***. Thus, in this study the value of *r* = 0.6 SD was used (Pincus, 1991; Richman and Moorman, 2000).

### Correlation Matrices

Pearson correlation was calculated between 7 pre-identified brain networks (Yeo et al., 2011) and between cortical and subcortical regions (42 cortical regions and 21 subcortical regions) of the brain (Harvard-Oxford mask FSL) across scans. This was done in both rest and task entropy maps, to identify the dependence of networks and regions on each other in rest and task mode.

In the case of the seven functional networks, *X* and *Y* were length 200 vectors representing the mean sample entropy of a network in each scan. Correlation was then calculated for each pair of networks and placed into a 7 × 7 correlation matrix representing the correlation between each network and every other network. This was calculated for both for rest and task sample entropy maps.

The same process was repeated for the 63 cortical and subcortical regions, where *X* and *Y* represented length 200 vectors representing the mean sample entropy of a distinct subcortical or cortical ROI. This results in a 63 × 63 correlation matrix of both cortical and subcortical regions.

### Paired *t*-Test and Reliability Evaluation

The paired *t*-test was used to compare brain entropy maps at rest and during the working memory task. This was performed at voxel-level resolution to produce a whole brain volume *t*-test, as well as for the seven networks and the cortical/subcortical regions. In every case, the two paired samples are length 200 vectors representing entropy in each scan at rest and task. Due to multiple comparison, the Bonferroni correction was done to decrease the risk of a type I error. Box plots were produced for the seven networks as well as the subcortical and cortical regions to further highlight differences.

In order to examine the reproducibility of the entropy measurements, we calculated them separately for the first and second rsfMRI scans for each subject, and for the first and second fMRI scans for each subject. We then measured correlation between the values for the seven networks across individuals in the two scans. We also measured correlation between the final group level values for the seven networks across the two scans.

The intraclass correlation (ICC) was also used to evaluate the reliability of the entropy calculation of each functional network by comparing the variability of entropy of the two scans of the same subject to the total variation across all subjects (for resting state and for task performing) using a Microsoft Excel Add-in to calculate ICC (3,1) (Merisaari et al., 2019).

## Results

**FIGURE 1:**
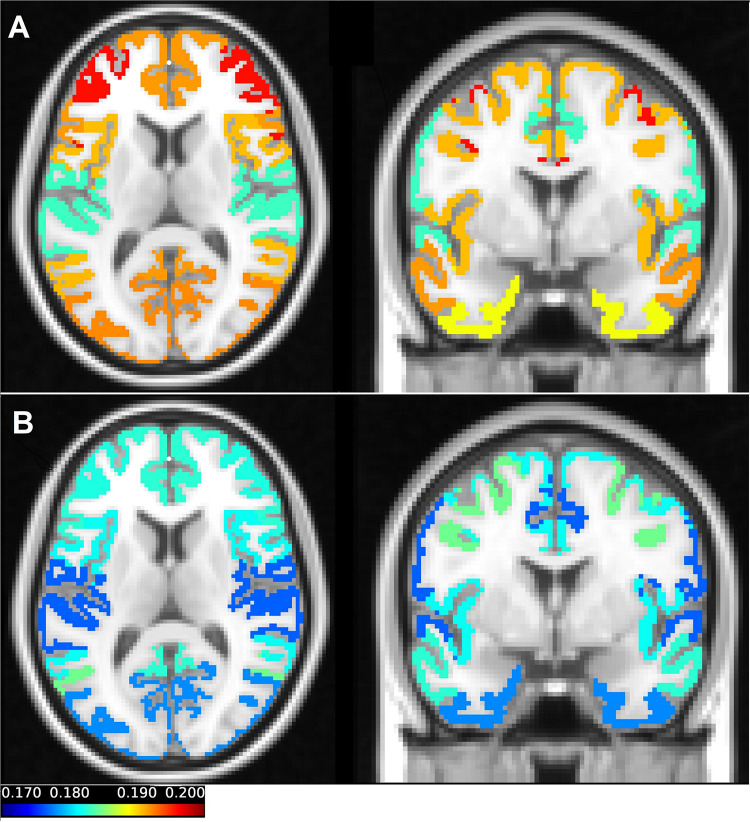
The average entropy values across scans of each of the seven networks in resting state **(A)** and during a working memory task **(B)** were mapped on the T1-weighted image. The coronal and axial views were selected in a way that all parcellations can be presented in one figure. In **(A)** the axial view clearly demonstrates the frontoparietal network, which has the highest value of the entropy during resting state. The somatomotor and limbic networks have low values of the entropy in the resting state. In **(B)** the dorsal attention network which has the highest entropy during the working memory task can be seen both in the coronal and axial view. Also, in **(B)** the visual and limbic networks are presented, which have the lowest values of entropy in task.

The sample entropy was calculated at each voxel in all fMRI task and rest scans (100 subjects, each of them scanned twice at rest and twice during task performance). Figure 1 shows the sample entropy network-level maps of resting-state (Figure 1A) and task-performing (Figure 1B) groups, superimposed onto the T1-weighted image. As shown in the figure, the frontoparietal network has the highest entropy during rest, while the dorsal attention network has the highest entropy during task. The somatomotor network has the lowest entropy during rest, also the limbic network and somatomotor have the lowest entropy during task. Entropy is generally higher during rest than during task.

**FIGURE 2:**
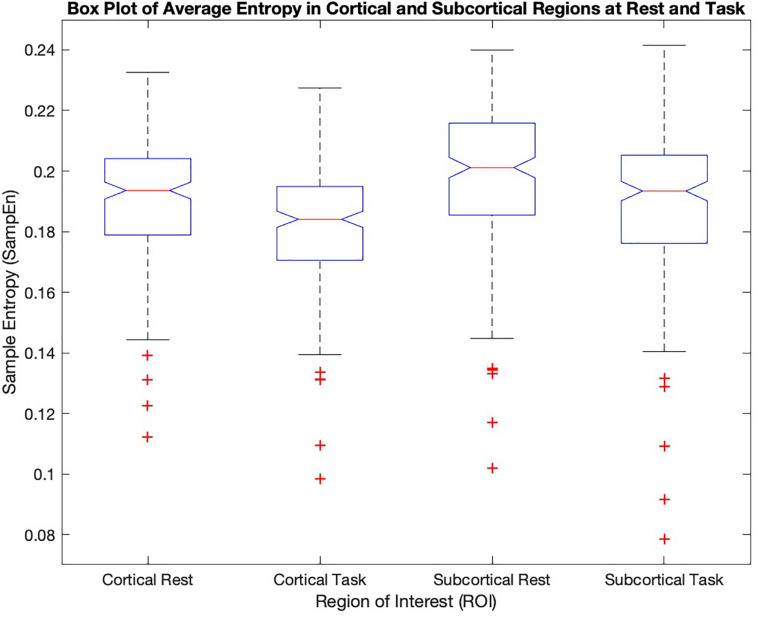
The mean sample entropy of cortical regions and subcortical regions at rest and during the working memory task are shown. Subcortical regions have higher significantly higher entropy than cortical regions (*p*-value < 0.05) both in task and at rest. The cortical and subcortical regions in rest show significantly higher values compared to task (*p*-value < 0.05).

Figure 2 shows quantitative values of Sample entropy for rest and task, divided into cortical and subcortical regions. The cortical regions in task have the average value of 0.1815 ± 0.0195. The average value of Sample entropy of cortical regions in rest is 0.1912 ± 0.0200, which is significantly higher than the same regions during the working memory task (*p*-value < 0.05). The subcortical regions in rest have the average value of 0.1981 ± 0.0232. The average value of Sample entropy for subcortical regions in task is 0.1897 ± 0.0245, which is significantly lower than the values of the same regions (subcortical) in rest. Interestingly, the subcortical regions during task show the widest spread of entropy values (standard deviation of 0.0245). Table 1 shows the average value and standard deviation of cortical and subcortical regions in rest and during task performance.

**TABLE 1:** The mean and standard deviations of cortical and subcortical regions at rest and during the working memory task.

**Region**	**Rest vs task**	**Mean**	**Standard deviation**
Cortical	Rest	0.1912	0.0200
	Task	0.1815	0.0195
Subcortical	Rest	0.1981	0.0232
	Task	0.1897	0.0245

**FIGURE 3:**
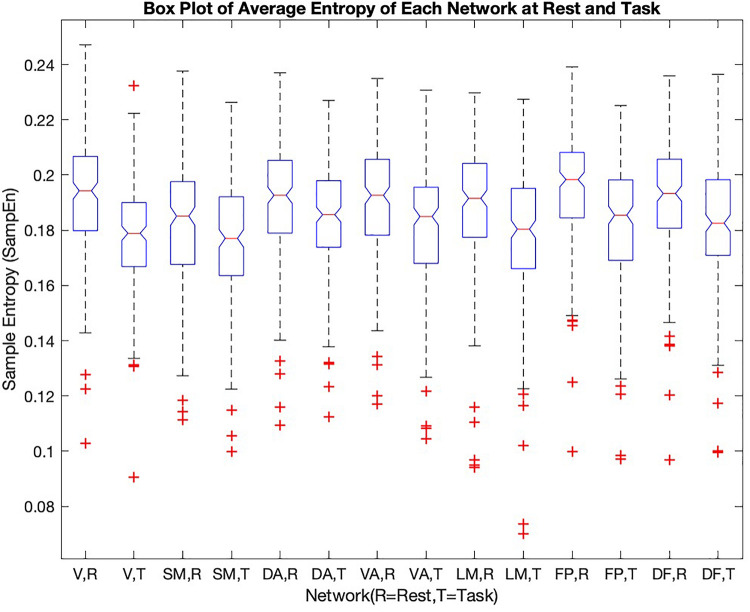
He sample entropy value of each of the seven cortical networks [1-visual (V), 2-somatomotor (SM), 3-dorsal attention (DA), 4-ventral attention (VA), 5-limbic (LM), 6-frontoparietal (FP), 7-default (DF)] in rest and during the working memory task are shown in the box plot (R = rest, T = task). The mean sample entropy for each network in rest is significantly higher than during the working memory task (*p*-value < 0.05, see Table 2)

To further examine the variability of entropy across brain networks, the values were separated by network (Figure 3).

The frontoparietal network has the highest entropy among all the cortical networks at rest. The somatomotor network with 0.1830 ± 0.0223 and limbic network with 0.1884 ± 0.0237 have the lowest entropy during rest. For each network, entropy during rest was significantly higher than during task (*p*-value is shown in the last column of Table 2).

**TABLE 2:** Means and standard deviations of all seven cortical networks at rest and during working memory task.

	**Network name**	**Rest vs task**	**Mean**	**Standard deviation**	***p*-Value**
1	Visual	Rest	0.1922	0.0212	1.01E-23
		Task	0.1779	0.0186	
2	Somatomotor	Rest	0.1830	0.0223	2.09E-33
		Task	0.1766	0.0239	
3	Dorsal attention	Rest	0.1908	0.0202	5.67E-29
		Task	0.1845	0.0203	
4	Ventral attention	Rest	0.1906	0.0212	7.86E-33
		Task	0.1816	0.0225	
5	Limbic	Rest	0.1884	0.0237	1.31E-24
		Task	0.1778	0.0241	
6	Frontoparietal	Rest	0.1958	0.0195	2.07E-29
		Task	0.1821	0.0221	
7	Default mode	Rest	0.1917	0.0200	3.28E-30
		Task	0.1825	0.0217	

During the working memory task, the dorsal attention network has the highest entropy. The limbic network, somatomotor network, and visual network have the low values of entropy during task performance.

**FIGURE 4:**
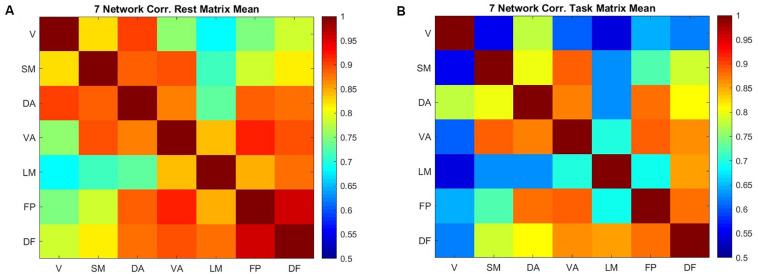
The correlation between the mean value of sample entropy for seven cortical networks across 200 scans (100 subjects) in resting state **(A)** and performing a working memory task **(B)**.

Correlation between the entropy of the networks describes how entropy in different areas covaries across subjects (Figure 4). This correlation is generally high but decreases during the task in comparison to the resting state. The frontoparietal network and default mode network have the highest correlation in the resting state (0.95), followed by the frontoparietal network and ventral attention network (0.92). During the working memory task, entropy in the ventral attention network is strongly correlated with entropy in both the frontoparietal network and the somatomotor networks (0.89). The dorsal attention network and frontoparietal network are nearly as strongly correlated (0.88). The lowest correlation is between the limbic network and the visual network, both at rest and during the working memory task. In task, the correlation between the visual network and the somatomotor network is among the lowest correlations (0.55), which is different from the resting state.

**FIGURE 5:**
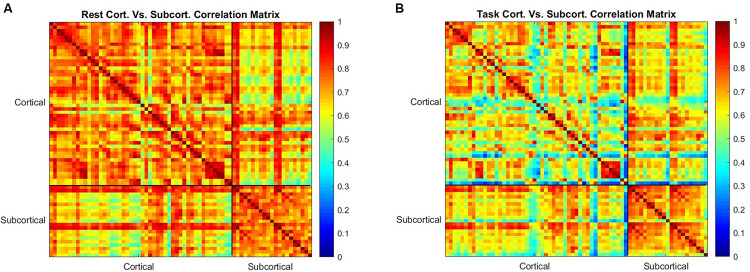
The correlation between the mean value of sample entropy for 42 cortical and 21 subcortical regions of interest as a measure of similarity in rest is shown in **(A,B)** shows the correlation between the same regions during the working memory task. Correlation is generally reduced during task performance, but the subcortical regions are less affected than the cortical regions.

We furthered examined the correlation of entropy values across the 42 cortical regions and 21 subcortical regions in rest and task (Figure 5). Correlation of entropy values is generally high within the cortical regions (average of 0.83) and within the subcortical regions (average of 0.89), but the correlation between cortical and subcortical regions is not as strong (average of 0.43). Also, by comparison of Figures 5A,B it can be observed that correlation between all ROIs is decreased during task, but this reduction in task mode is more severe in the cortical regions.

To determine the significance of the effects we observed, a two-factor ANOVA test was performed with the null hypotheses that

1- performing the memory task has no significant effect on the entropy of the brain (comparing ROI voxels)
2- brain regions (Cortical/subcortical) has no significant effect on the entropy of the brain.

As it is presented in Table 3, both the differences across rest and task and across cortex and subcortex were significant (*p*-value < 0.05), but there was no significant interaction effect. We performed a similar analysis on the data separated by network during rest and task.

**TABLE 3:** Two-factor ANOVA comparing mean cortical/subcortical entropies in 200 scans at task and rest.

**Source**	**Sum of squares**	**d.f.**	**Mean square**	**F score**	***p*-Value**
Rest/task	0.0164	1	0.0164	34.2164	7.1983e-09
Cortical/ subcortical	0.0115	1	0.0115	23.9758	1.1815e-06
Interaction	8.7399e-05	1	8.7399e-05	0.1824	0.6695
Error	0.3815	796	4.7928e-04		
Total	0.4095	799			

The two null hypotheses were:

1- performing the working memory task has no significant effect on the entropy of the brain
2- brain cortical networks have no significant effect on the entropy of the brain.

As it is presented in Table 4, significant differences were observed between rest and task and across networks (*p*-value < 0.05). Moreover, the interaction term was significant, indicating that task performance affects entropy in a network-dependent manner.

**TABLE 4:** Two-factor ANOVA comparing mean network entropies in 200 scans at task and rest.

**Source**	**Sum of squares**	**d.f.**	**Mean square**	**F score**	***p*-Value**
Rest/task	0.0691	1	0.0691	148.3814	2.7085e-33
Networks	0.0231	6	0.0039	8.2657	6.8201e-09
Interaction	0.0062	6	0.0010	2.2006	0.0402
Error	1.2980	2786	4.6589e-04		
Total	1.394	2799			

Table 5 presents the consistency of entropy measurements across scans. Pearson correlation ranges from 0.46 to 0.59 for rest and from 0.36 to 0.47 for task. Interestingly, entropy values are more consistent across scans in rest than in task. The means of scan 1 and 2 within each network are not significantly different (α = 0.05).

**TABLE 5:** Consistency of entropy values in seven functional networks for scan 1 and scan 2.

	**Rest**			**Task**		
	**ICC**	***r***	***p* Two-tail**	**ICC**	***r***	***p* Two-tail**
Visual	0.454	0.456	0.883	0.429	0.432	0.845
Somatomotor	0.474	0.475	0.092	0.467	0.468	0.915
Dorsal attention	0.513	0.523	0.755	0.365	0.369	0.321
Ventral attention	0.586	0.587	0.834	0.454	0.466	0.177
Limbic	0.525	0.527	0.660	0.345	0.363	0.107
Frontoparietal	0.554	0.556	0.464	0.370	0.382	0.174
Default mode	0.535	0.539	0.700	0.381	0.400	0.377

The ICC values are shown in Table 5 as well. Zuo et al. (2019) and Xing and Zuo (2018) categorized the ICC values in to the following intervals 0 < ICC < 0.2 (slight), 0.2 < ICC < 0.4 (fair), 0.4 < ICC < 0.6 (moderate), 0.6 < ICC < 0.8 (substantial), 0.8 < ICC < 1.0 (almost perfect) for reliability quantification. By considering those intervals, all seven networks can be categorized as having moderate reliability in resting state. In task, subjects’ visual network, somatomotor network and ventral attention network are categorized within moderate reliability and dorsal attention network, limbic network, frontoparietal network, and default mode network are categorized within fair reliability.

At the group level, the measurements are quite consistent. Values are given in Table 6. The correlation between average entropy values for the seven networks across the two scans is 0.96 during rest, and 0.87 during task. The lower group-level correlation during task is consistent with the reduced consistency during task observed at the individual level.

**TABLE 6:** The average entropy and standard deviation of each network for scan 1 and scan 2.

	**Rest**				**Task**			
	**Scan 1 ($\bar{x}$)**	**Scan 1 (σ)**	**Scan 2 ($\bar{x}$)**	**Scan 2 (σ)**	**Scan 1 ($\bar{x}$)**	**Scan 1 (σ)**	**Scan 2 ($\bar{x}$)**	**Scan 2 (σ)**
Visual	0.19235	0.02226	0.19202	0.02018	0.1781	0.0174	0.1777	0.0198
Somatomotor	0.18497	0.02280	0.18109	0.02171	0.1765	0.0231	0.1767	0.0247
Dorsal attention	0.19045	0.02187	0.19108	0.01849	0.1857	0.0189	0.1834	0.0217
Ventral attention	0.19084	0.02180	0.19043	0.02078	0.1832	0.0198	0.1800	0.0249
Limbic	0.18790	0.02287	0.18892	0.02466	0.1800	0.0199	0.1756	0.0276
Frontoparietal	0.19515	0.02043	0.19650	0.01854	0.1838	0.0192	0.1804	0.0247
Default mode	0.19129	0.02115	0.19204	0.01880	0.1836	0.0182	0.1814	0.0248

## Discussion

In this article sample entropy was used to quantify the temporal complexity of fMRI data. The complexity of time-series obtained from healthy subjects in resting state and during the performance of a working memory task were calculated. Furthermore, Pearson correlation was calculated to examine the similarity between the complexities of different brain networks across individuals.

Our results add to existing studies of complexity in the BOLD signal to show that entropy varies across brain networks and during working memory as compared to rest. In general, entropy decreases during a task in a network-dependent manner. Moreover, when examined across subjects, entropy tends to change in the same way across many brain areas, giving rise to strong correlations within subcortical regions and within subcortical regions. Interestingly, the correlation is weaker between subcortical and cortical regions, suggesting that while entropy within each region tends to change in the same way across subjects, there is greater variability in the relation between the two regions at the individual level.

### Entropy During Rest and Task Performance

Entropy across the brain has an average value of 0.1913 ± 0.023 (in rest) and 0.1815 ± 0.019 (in working memory task), indicating that it falls within a fairly narrow range. Within this range, however, there are distinct variations across networks and between cortical and subcortical structures. The cortical areas both in the task data and rest data demonstrate significantly lower entropy in comparison to the subcortical areas. These findings are consistent with previous work by Jia et al. (2017), who created a sample entropy map of the brain in healthy subjects and showed higher values in the caudate, the olfactory gyrus, the amygdala, and the hippocampus, and lower values in primary sensorimotor and visual areas. If entropy of the BOLD signal is taken as a surrogate for neural complexity, this indicates that the neural activity in the cortical areas is more organized than in subcortical areas, in line with the general view of the cortex as the primary site of most cognitive processes. The decrease in entropy in both cortical and subcortical areas during the working memory task might then reflect an increase in the coordination of activity needed to perform the task. Interestingly, the decrease in entropy was not limited to areas typically activated by the task. For example, the extensive activation during the task is observed in the frontoparietal network (Barch et al., 2013), but comparable changes in entropy are found in networks like the limbic network that are not typically activated. Moreover, the default mode network is deactivated during task performance, so that if entropy directly reflected activity, entropy there should increase during the task. In fact, we observe a decrease in entropy instead, evidence that entropy is sensitive to aspects of the BOLD signal that are not directly tied to activity levels. These findings are consistent with a previous study by Zhang et al. (2016), who observed differences in entropy across fMRI studies while subjects were listening passively to (i) emotionally neutral words alternating with no word as the control condition (neutral-blank), and (ii) threat-related words alternating with emotionally neutral word as the experimental condition (threat-neutral). The relative independence of entropy measures from changes in the BOLD signal associated with activation during a task suggests they may provide complementary insight into brain function.

### Similarity Across Networks

In both rest and task, the entropy of both the visual network and the limbic network tends to be less coupled to other brain networks across individuals. For the most part, the relationship between networks is similar across rest and task. However, during the working memory task, the entropy in the dorsal attention network becomes less coupled to entropy in the visual and somatomotor networks. When the brain is further divided into 69 parcels, the decoupling between areas is especially noticeable in the cortex, where there are clear differences in the amount of change in correlation between particular cortical areas during the task. This suggests that there may be an interesting amount of individual variability that relates to task performance, and future work should examine whether particular patterns of entropy changes during task predict performance on the task by the individual subject.

### Limitations

As with all BOLD-based measurements, the entropy calculated here is based on the hemodynamic response to neural activity rather than the activity itself. The inherent lowpass filter of the vasculature limits the frequency content of the signal and reduces the amount of information it carries. However, the change in entropy observed during task performance is promising evidence that some of the information about the complexity of neural activity is preserved in the BOLD signal.

## Conclusion

BOLD-based measurements of sample entropy vary across brain regions, with lower values in cortical than subcortical areas. During performance of a working memory task, entropy decreases across the whole brain but in a region-dependent manner. Both of these findings are consistent with the idea that entropy encodes information about the complexity of neural activity that is separate from simple measurements of activation. When examined across individuals, entropy changes are generally correlated, particularly within cortical and within subcortical areas. More variability in this correlation is observed during the working memory task, hinting at potentially important differences at the subject level that may be linked to performance.

## Data Availability Statement

The datasets generated for this study are available on request to the first author.

## Author Contributions

HT and MN performed the data preprocessing and analysis. SK and MN performed the results interpretation, comparison of obtained results with existing materials, preparation of document. All authors contributed to the article and approved the submitted version.

## Conflict of Interest

The authors declare that the research was conducted in the absence of any commercial or financial relationships that could be construed as a potential conflict of interest.

## Footnotes

1 https://cfn.upenn.edu/~zewang/BENtbx.php
